# Association between phosphate and long-term outcome in CAD patients underwent coronary intervention

**DOI:** 10.1038/s41598-021-99518-z

**Published:** 2021-10-11

**Authors:** Tsung-Ying Tsai, Pai-Feng Hsu, Cheng-Hsueh Wu, Ya-Ling Yang, Su-Chan Chen, Shao-Sung Huang, Wan Leong Chan, Shing-Jong Lin, Jaw-Wen Chen, Ju-Pin Pan, Min-Ji Charng, Ying-Hwa Chen, Tao-Cheng Wu, Tse-Min Lu, Po-Hsun Huang, Hao-Min Cheng, Chin-Chou Huang, Shih-Hsien Sung, Yenn-Jiang Lin, Hsin-Bang Leu

**Affiliations:** 1 Division of Cardiology, Department of Medicine, Taipei Veterans General Hospital, Taipei, Taiwan; 2 Healthcare and Management Center, Taipei Veterans General Hospital, 201, Sec. 2, Shih-Pai Road, Taipei, Taiwan; 3 Faculty of Medicine, National Yang-Ming University, Taipei, Taiwan; 4 Institute of Clinical Medicine and Cardiovascular Research Center, National Yang-Ming University, Taipei, Taiwan

**Keywords:** Cardiology, Interventional cardiology

## Abstract

Phosphate has been linked to higher cardiovascular (CV) risk. However, whether phosphate is associated with poor outcomes for patients with coronary artery disease (CAD) after percutaneous coronary interventions (PCIs) remained undetermined. 2,894 CAD patients (2,220 male, aged 71.6 ± 12.2), who received PCI at TVGH from 2006 to 2015, with phosphate measurement, were enrolled. The primary outcome was the composite of major adverse CV events [MACE, comprising of CV death, nonfatal MI, and nonfatal stroke] and heart failure hospitalization (HHF). The key secondary outcome was MACE. There was a J-curve association between phosphate and CV events after adjusted for comorbidities and renal function. Phosphate around 3.2 ± 0.1 mg/dL was associated with the lowest CV risk. In Cox analysis, each 1 mg/dL increases in phosphate was associated with a higher risk of MACE + HHF (HR: 1.12, 95% CI: 1.05–1.21): CV death (HR: 1.37, 95% CI: 1.22–1.55) and HHF (HR: 1.12, 95% CI: 1.02–1.23). Subgroup analyses showed more prominent association between phosphate and MACE + HHF in male, age > 65, bare-metal stents (BMSs), LVEF < 50%, eGFR < 60, LDL > 70 mg/dL, and emergent PCI. Phosphate has a significant association with the risk of CV events in CAD patients undergoing PCI that was independent of comorbidities and renal function.

## Introduction

The serum phosphate level is tightly regulated in order to maintain various aspects of human physiology, including energy metabolism, neurotransmission, endothelial function, bone formation, and cardiovascular (CV) function^1^. Although abnormal phosphate metabolism is considered the hallmark of chronic kidney disease (CKD), studies have shown that abnormal serum phosphate levels are associated with vascular calcification, atherosclerosis, all-cause, and CV mortality, regardless of renal function^2–4^.

Phosphate was also reported to take part in the pathogenesis of the CV disease process^5^. Hormone and markers related to the regulation of phosphate metabolism, such as serum parathyroid hormone (PTH), alkaline phosphatase (ALKP), fibroblast growth factor 23 (FGF-23), and 1.25-(OH)2-D3 (VitD3) levels as all being linked to the development and progression of CV disease through vascular calcification or systemic inflammation^6–8^. Percutaneous coronary intervention (PCI) is the main method of coronary revascularization. However, the prognosis of patients with CKD undergoing PCI is unsatisfactory^9–12^. Previous studies have shown that increased serum levels of phosphate were associated with poor revascularization outcomes^13^. However, they only investigated patients undergoing regular dialysis with a short follow-up period. Furthermore, there is limited information about the interaction between serum phosphate and future adverse risk in situations, such as the choice of stent type, the underlying left ventricular ejection fraction (LVEF), and whether the patient presented with acute coronary syndrome (ACS). As such, we conducted this retrospective Asian CAD cohort study to assess the association of serum phosphate and future CV events in CAD patients with PCI.

## Methods

We retrospectively reviewed patients who had received PCI for CAD from 2006 to 2015 at the Taipei Veterans General Hospital. Patients with a measured serum phosphate level at enrollment, who underwent successful PCI, were enrolled. The demographic characteristics, biochemical data, procedural details, and clinical outcomes of these patients were collected from the electronic medical record review. This study followed the Declaration of Helsinki and was approved by the Internal Research Board of Taipei Veterans General Hospital (IRB No. 2016-03-014CC).

It was not appropriate or possible to involve patients or the public in the design, or conduct, or reporting, or dissemination plans of our research. The patient data used in this study was untraceable and anonymous before entering the analysis. According to the Internal Research Board of Taipei Veterans General Hospital, patient informed consent formed was not required.

PCI procedures were performed in conformity with the 2010 ESC/EACTS guidelines on myocardial revascularization^14^. In brief, coronary angiography was performed with standard procedures. Unfractionated heparin was administered to achieve an activated clotting time of > 300 s. After successful wire-crossing, lesion modification was usually performed by balloon dilatations. Rotablations were performed for heavily calcified or balloon-uncrossable lesions. Following dilatation and/or lesion modification, a stent was deployed for most lesions. Successful PCI was defined as residual stenosis < 30% with thrombolysis in MI (TIMI), grade 3 flow at the end of the procedure. Following the procedures, all patients received aspirin (100 mg/d) indefinitely and a P2Y12 inhibitor for at least one month if a bare-metal stent (BMS) was deployed, or at least one year if a drug-eluting stent (DES) was deployed. All patients were observed for a minimum of 8 h and then discharged under stable conditions.

Baseline information such as body mass index (BMI) and smoking status were collected. We obtained a detailed medical history for comorbid conditions such as hypertension (HTN), diabetes mellites (DM), dyslipidemia, stroke, chronic kidney disease (CKD), heart failure (HF), and the medication history transcribed from electronic medical records. Procedure details of the PCI, including indication for the procedure [acute coronary syndrome (ACS) or elective), the type of deployed stents [bare metal stent (BMS) or drug eluting stent (DES)], were collected from the procedure notes. ACS procedures were defined as procedures arranged for acute ST-elevation myocardial infarction (STEMI), Non–ST-elevation myocardial infraction (NSTEMI) and Unstable angina. All the non-emergent procedures were classified as elective. Biochemical parameters, including serum phosphate, serum calcium, serum creatinine, uric acid, hemoglobin, low-density lipoprotein (LDL), and high-density lipoprotein (HDL), were measured using a TBA-c16000 automatic analyzer (Toshiba Medical Systems, Tochigi, Japan) following an overnight fast before the index procedure. The ejection fraction from the left ventriculography and echocardiographic study were also evaluated when available.

The primary endpoint was the composite of CV deaths, nonfatal MIs, nonfatal strokes, or HF hospitalizations. The key secondary outcome was major adverse CV events [MACE, defined as the composite of CV deaths, nonfatal MIs, and nonfatal strokes]. Other secondary outcomes included the individual components of MACE, HF hospitalizations, and repeat revascularization. CV death was defined as deaths that result from an MI, sudden cardiac death, death due to HF, death due to stroke, death due to CV procedures, death due to CV hemorrhage, and death due to other CV causes. MI was defined by the in-charge cardiologist according to the third definition of MI. Stroke was defined as the combination of ischemic and hemorrhagic stroke. Heart failure hospitalization was defined as any hospitalization with a primary diagnosis of HF or with one of the first two secondary diagnoses being HF.

The data was expressed as mean ± standard deviation for continuous variables and mean ± 95% confidence interval (CI) for categorical variables. Demographic characteristics and biochemical variables were compared using the Student’s t-test to compare continuous variables when appropriate, with chi-square tests used for categorical variables. The association between the serum phosphate level and clinical and biochemical variables was evaluated sequentially with multiple linear regression. To evaluate the association between serum phosphate and clinical outcomes, the serum phosphate was categorized into four quartiles. Survival to the primary and secondary endpoints of the four groups was compared with the stepwise Cox proportional hazards models, while backward selection was used to calculate hazard ratios (HRs) and 95% CI for serum phosphate categories. To adjust for confounding variables, a second Cox hazard ratio was performed with adjustment for age, gender, hypertension, DM, smoking status, and estimated glomerular filtration rate (eGFR). Subgroup and sensitivity analyses were also performed. Prespecified subgroups in these analyses were defined according to age (< 65 years of age, or > 65 years of age or older), gender, DM, hypertension (HTN), smoking, BMI (< 22 kg/m^2^, or 22 kg/m^2^ or more), LDL (< 70 mg/dL, 70 mg/dL or more), HDL (< 40 mg/dL, 40 mg/dL or more), ACS, eGFR (< 60 ml/min/1.73^2^, 60 ml/min/1.73^2^ or more), LVEF (< 50%, 50% or more), DES use, and dialysis status. Statistical significance was set as *p* < 0.05. All statistical analyses were carried out with SPSS 20.0 software (IBM, Inc. Chicago, IL, USA).

## Results

### Patient demographics

Among 8,794 consecutive patients who received PCI for CAD between 2006 to 2015, a total of 2,894 patients (2,220 male, aged 71.6 ± 12.2) with a baseline phosphate value recorded were enrolled. The average follow-up duration was 65.1 ± 32.1 months. The demographic characteristics of the participants are shown in Table 1. Serum phosphate ranged from 0.5 to 12.1 mg/dL (mean 3.6 ± 1.1 mg/dL). There were 270 (9.3%) patients with hypophosphatemia, defined as a phosphate level < 2.5 mg/dL while 356 (12.3%) patients had hyperphosphatemia, defined as phosphate level > 4.5 mg/dL. Patients with a higher phosphate level were more likely to be younger and female. They also had a higher prevalence of DM and CKD, demonstrated in Table 1. Yet, patients within the second quartile of phosphate levels tended to have higher eGFR, lower serum calcium, and a lower prevalence of heart failure. There was a J-curved association between the eGFR and serum phosphate levels, as shown in supplement Fig. 1. In linear correlation analysis, age, female gender, HF, CKD, serum uric acid, calcium, and creatinine were significantly associated with serum phosphate levels, shown in supplement Table 1.

**Table 1:** Baseline clinical characteristics among quartiles of serum phosphate.

	Overall (n = 2894)	1st quartile (IP 0 ~ 3 mg/dL) (n = 765)	2nd quartile (IP 3 ~ 3.4 mg/dL) (n = 790)	3rd quartile (IP 3.5 ~ 4 mg/dL) (n = 762)	4th quartile (IP > 4 mg/dL) (n = 667)	*p* value	*p* for trend
IP	3.56 ± 1.08	2.49 ± 0.41	3.21 ± 0.14	3.72 ± 0.17	5.01 ± 1.16	<0.0001	<0.0001
Male gender (n, %)	2220 (74.4)	627 (82.0)	639 (80.9)	559 (73.4)	395 (59.2)	< 0.001	<0.0001
Age (y/o)	71.59 ± 12.20	74.54 ± 11.20	71.28 ± 12.00	71.16 ± 12.52	69.08 ± 12.52	< 0.001	<0.0001
BMI	25.06 ± 3.98	24.69 ± 3.92	25.24 ± 3.98	25.10 ± 3.74	25.21 ± 4.27	0.040	0.031
Smoking (n, %)	1149 (38.5)	314 (41.1)	317 (40.1)	296 (38.9)	222 (33.3)	0.0136	0.004
Dyslipidemia (n, %)	696 (23.3)	115 (15.0)	95 (12.0)	78 (10.2)	84 (12.6)	0.0413	<0.0001
Diabetes (n, %)	1341 (44.9)	298 (39.0)	336 (42.5)	337 (44.2)	370 (55.5)	<0.0001	<0.0001
Hypertension (n, %)	2543 (85.2)	653 (85.4)	682 (86.3)	647 (84.9)	561 (84.1)	0.6829	0.403
Heart failure (n, %)	593 (19.9)	148 (19.4)	124 (15.7)	141 (18.5)	180 (27.0)	<0.0001	<0.0001
Stroke (n, %)	230 (7.7)	52 (6.8)	47 (6.0)	75 (9.8)	56 (8.4)	0.0218	0.028
CKD (n, %)	351 (11.8)	66 (8.6)	67 (8.5)	69 (9.1)	149 (22.3)	<0.0001	<0.0001
ACS (n, %)	1318 (44.2)	144 (18.8)	178 (22.5)	163 (21.4)	153 (22.9)	0.929	0.654
DES (n, %)	1517 (50.8)	340 (44.4)	417 (52.8)	383 (50.3)	377 (56.5)	0.001	0.197
Creatinine	2.06 ± 2.23	1.63 ± 1.48	1.59 ± 1.40	1.69 ± 1.66	3.39 ± 3.35	<0.0001	<0.0001
eGFR	53.20 ± 29.34	56 ± 24	59.14 ± 29.76	57.04 ± 28.21	38.17 ± 30.38	<0.0001	<0.0001
Hemoglobin	12.23 ± 2.12	12.34 ± 2.05	12.64 ± 2.05	12.44 ± 2.01	11.38 ± 2.19	<0.0001	<0.0001
Uric acid	6.60 ± 2.08	6.19 ± 2.01	6.49 ± 1.96	6.66 ± 1.99	7.13 ± 2.30	<0.0001	<0.0001
Calcium	8.90 ± 0.72	9 ± 0.76	8.91 ± 0.61	8.96 ± 0.69	8.97 ± 0.87	<0.0001	<0.0001
HDL	42.20 ± 12.47	41.87 ± 12.34	43.10 ± 12.53	42.29 ± 12.59	41.38 ± 12.36	0.070	0.3337
LDL	104.10 ± 34.76	102.11 ± 35.24	103.34 ± 32.51	107.06 ± 34.72	103.78 ± 36.70	0.042	0.053
Beta blockers	1246 (41.8%)	289 (37.8%)	329 (41.7%)	336 (44.1%)	292 (43.8%)	0.05	0.011
Statins	1386 (46.4%)	327 (42.8%)	376 (48.0%)	402 (52.8%)	281 (42.1%)	< 0.0001	0.489
ACEI/ARB	1378 (46.2%)	348 (45.5%)	386 (48.9%)	338 (44.4%)	306 (45.9%)	0.099	0.245
Thiazide diuretics	313 (10.8%)	76 (9.9%)	89 (11.3%)	76 (10.0%)	72 (10.8%)	0.820	0.968

ACEI/ARB = angiotensin converting enzyme inhibitor/angiotensin receptor blockers; DES = drug eluting stent; CKD = chronic kidney disease, HDL = high density lipoprotein-cholesterol; LDL = low density lipoprotein-cholesterol.

### Clinical events

**Figure 1:**
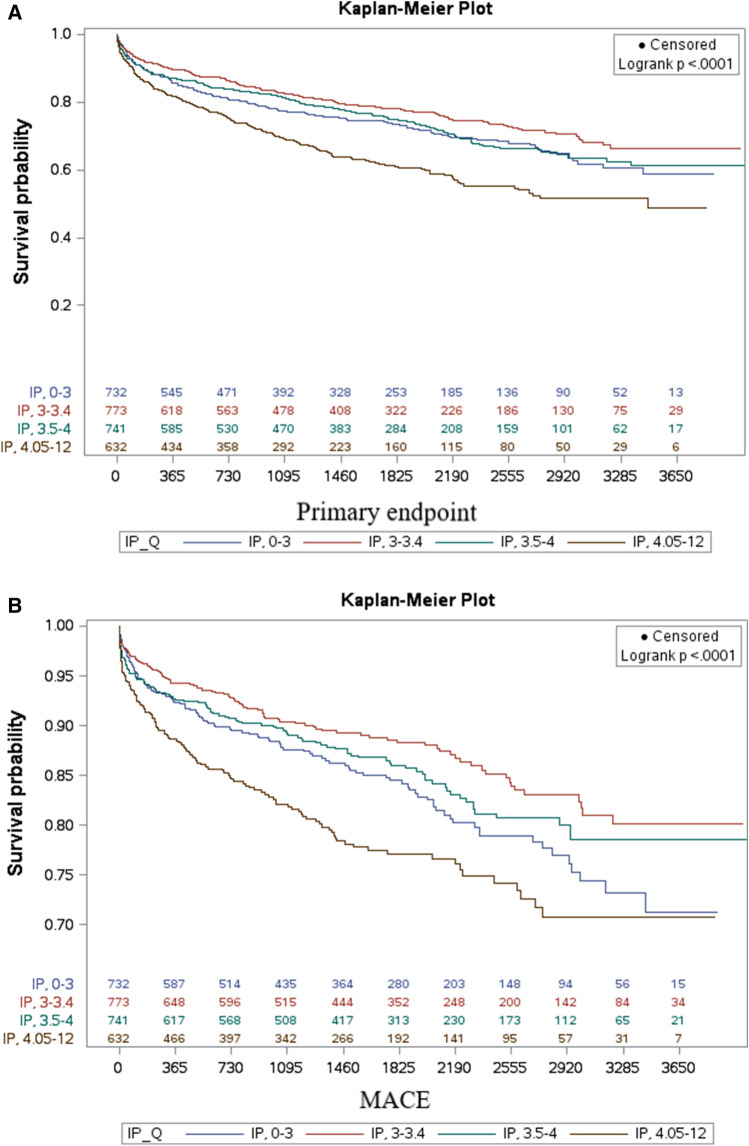
(**A**) The Kaplan–Meier curve for survival to the primary outcome divided by quartiles of serum phosphate level. *p* values are for the overall comparison among the groups using the log rank test. IP = serum phosphate. (**B**) The Kaplan–Meier curve for survival to MACE divided by quartiles of serum phosphate level. *p* values are for the overall comparison among the groups using the log rank test. MACE = major adverse cardiovascular events.

**Figure 2:**
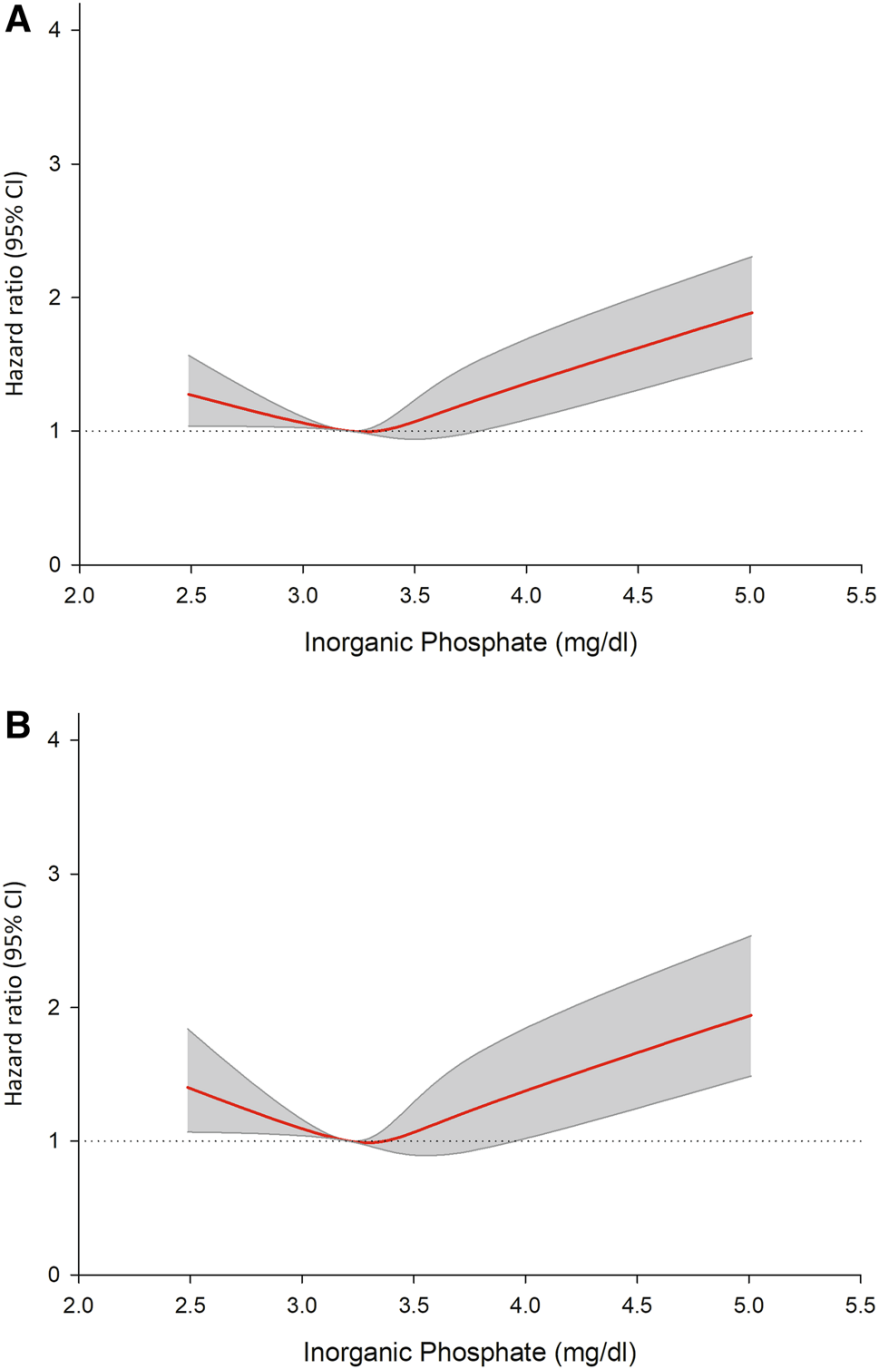
(**A**) Cubic spline analysis. The plot describes the relationship between serum phosphate as a continuous variable and the probability of the primary endpoint. The grey area indicates the 95% confidence interval. (**B**) Cubic spline analysis. The plot describes the relationship between serum phosphate as a continuous variable and the probability of MACE. The grey area indicates the 95% confidence interval. MACE = major adverse cardiovascular events.

During a median follow-up of 65.1 ± 32.1 months, 770 (26.6%) patients met the primary endpoint, while 443 (15.3%) patients met the key secondary endpoint of MACE. For the other secondary endpoints, there were 173 (6.0%) CV deaths, 91 (3.1%) nonfatal strokes, 211 (7.3%) nonfatal MIs, 416 (14.4%) HF hospitalizations, and 579 (20.0%) repeated revascularization procedures in the follow-up period. Compared with the 1st, 3rd_,_ and 4th quartiles of phosphate levels, patients within the second quartile phosphate level (between 3.0–3.4 mg/dL) had the lowest risk for the primary endpoint, MACE, CV death, and HF hospitalizations, demonstrated in Table 2. Kaplan–Meier curves and log-rank analyses also showed that patients in the second quartile of serum phosphate levels had a significantly lower risk for the primary endpoint and MACE (Fig. 1A,B). Spline curve analysis showed a J-shaped relationship between serum phosphate levels and the odds of the primary endpoint, with MACE as the lowest point between phosphate levels of 3.0–3.4 mg/dL as well (Fig. 2A,B).

**Table 2:** Association between serum phosphate level in quartiles and clinical outcomes.

	Events, n (%)	Unadjusted (model 1)		Adjusted* (model 2)	
		HR (95% CI)	*p* value	HR (95% CI)	*p* value
**Primary endpoint**					
Q1 (0 ~ 3 mg/dL)	190 (24.8)	1.28 (1.04–1.57)	0.021	1.15 (0.93–1.41)	0.194
Q2 (3 ~ 3.4 mg/dL)	173 (21.9)	Reference	–	Reference	–
Q3 (3.5 ~ 4 mg/dL)	193 (25.3)	1.20 (0.98–1.47)	0.084	1.15 (0.94–1.41)	0.185
Q4 (> 4 mg/dL)	214 (32.1)	1.89 (1.54–2.31)	0.000	1.55 (1.25–1.92)	0.000
Cont	–	1.18 (1.11–1.26)	0.000	1.12 (1.05–1.21)	0.001
**MACE**					
Q1 (0 ~ 3 mg/dL)	116 (15.2)	1.40 (1.07–1.84)	0.015	1.31 (1.00–1.72)	0.053
Q2 (3 ~ 3.4 mg/dL)	95 (12.0)	Reference	–	Reference	–
Q3 (3.5 ~ 4 mg/dL)	107 (14.0)	1.21 (0.91–1.59)	0.185	1.16 (0.88–1.53)	0.292
Q4 (> 4 mg/dL)	125 (18.7)	1.94 (1.49–2.54)	0.000	1.53 (1.15–2.03)	0.004
Cont	–	1.21 (1.11–1.31)	0.000	1.13 (1.03–1.24)	0.007
**MI**					
Q1 (0 ~ 3 mg/dL)	54 (7.1)	1.39 (0.94–2.07)	0.101	1.37 (0.92–2.04)	0.118
Q2 (3 ~ 3.4 mg/dL)	45 (5.7)	Reference	–	Reference	–
Q3 (3.5 ~ 4 mg/dL)	50 (6.6)	1.18 (0.79–1.77)	0.419	1.15 (0.76–1.72)	0.510
Q4 (> 4 mg/dL)	62 (9.3)	2.11 (1.43–3.09)	0.000	1.51 (1.00–2.27)	0.049
Cont	–	1.22 (1.09–1.38)	0.001	1.07 (0.94–1.23)	0.298
**Stroke**					
Q1 (0 ~ 3 mg/dL)	29 (3.8)	1.54 (0.88–2.67)	0.129	1.47 (0.84–2.57)	0.177
Q2 (3 ~ 3.4 mg/dL)	22 (2.8)	Reference	–	Reference	–
Q3 (3.5 ~ 4 mg/dL)	25 (3.3)	1.22 (0.69–2.16)	0.505	1.21 (0.68–2.15)	0.521
Q4 (> 4 mg/dL)	15 (2.3)	1.06 (0.55–2.05)	0.861	1.03 (0.52–2.05)	0.935
Cont	–	0.98 (0.78–1.23)	0.842	0.99 (0.78–1.26)	0.934
**CV death**					
Q1 (0 ~ 3 mg/dL)	36 (4.7)	1.22 (0.76–1.97)	0.406	1.07 (0.66–1.73)	0.780
Q2 (3 ~ 3.4 mg/dL)	32 (4.1)	Reference	–	Reference	–
Q3 (3.5 ~ 4 mg/dL)	40 (5.3)	1.31 (0.82–2.09)	0.251	1.24 (0.78–1.97)	0.372
Q4 (> 4 mg/dL)	65 (9.8)	2.73 (1.79–4.17)	0.000	2.17 (1.39–3.39)	0.001
Cont	–	1.38 (1.25–1.53)	0.000	1.37 (1.22–1.55)	0.000
**HF**					
Q1 (0 ~ 3 mg/dL)	101 (13.2)	1.20 (0.91–1.59)	0.200	1.03 (0.78–1.36)	0.832
Q2 (3 ~ 3.4 mg/dL)	96 (12.2)	Reference	–	Reference	–
Q3 (3.5 ~ 4 mg/dL)	106 (13.9)	1.17 (0.89–1.54)	0.272	1.11 (0.84–1.46)	0.464
Q4 (> 4 mg/dL)	113 (16.9)	1.77 (1.35–2.33)	0.000	1.51 (1.13–2.01)	0.005
Cont	–	1.15 (1.05–1.26)	0.002	1.12 (1.02–1.23)	0.023
**Revascularization**					
Q1 (0 ~ 3 mg/dL)	148 (19.4)	1.05 (0.84–1.31)	0.676	1.09 (0.87–1.36)	0.476
Q2 (3 ~ 3.4 mg/dL)	160 (20.3)	Reference	–	Reference	–
Q3 (3.5 ~ 4 mg/dL)	163 (21.4)	1.09 (0.88–1.36)	0.435	1.09 (0.87–1.35)	0.449
Q4 (> 4 mg/dL)	108 (16.2)	0.97 (0.76–1.24)	0.831	0.93 (0.72–1.20)	0.568
Cont	–	1.00 (0.92–1.09)	0.977	0.97 (0.89–1.07)	0.541

Adjusted*(Model 2) was adjusted for Age, Gender, HTN, DM, Smoking and eGFR.

MI = myocardial infraction, HF = hospitalization for decompensated heart failure, MACE = major adverse cardiovascular events including cardiovascular deaths, nonfatal stroke and nonfatal MI.

Table 2 showed an association between serum phosphate values and future CV risks, according to quartiles (the second quartile was the reference category) and continuous values. The unadjusted Cox-regression (model 1 of Table 2) showed there was a higher risk for the primary endpoint: MACE, CV death, MI, and HF hospitalization for every 1 mg/dl increase in serum phosphate. The unadjusted model (model 1 of Table 2) also showed the risk of the primary endpoint, with MACE significantly higher in patients in the first and fourth quartiles of the phosphate levels, while MI, CV death, and HF hospitalization was significantly higher in patients within the fourth quartile of phosphate levels. However, the risk of ischemic stroke and repeat revascularization was not associated with serum phosphate levels. After adjustment for age, gender, HTN, DM, smoking, and eGFR, Cox-regression (model 2 of Table 2) showed that there was a higher risk of the primary endpoint (HR: 1.12, 95% CI:1.05–1.21, *p* = 0.001), MACE (HR: 1.13, 95% CI: 1.03–1.24, *p* = 0.007), CV death (HR: 1.37, 95% CI: 1.22–1.55, *p* < 0.001), MI (HR: 1.07, 95% CI: 0.94–1.23, *p* = 0.298), and HF-related hospitalization (HR: 1.12, 95% CI: 1.02–1.23, *p* = 0.023) for every 1 mg/dl increase in serum phosphate. However, the higher risk was only seen in patients with phosphate levels in the fourth quartile after adjustment.

### Subgroup analysis

**Figure 3:**
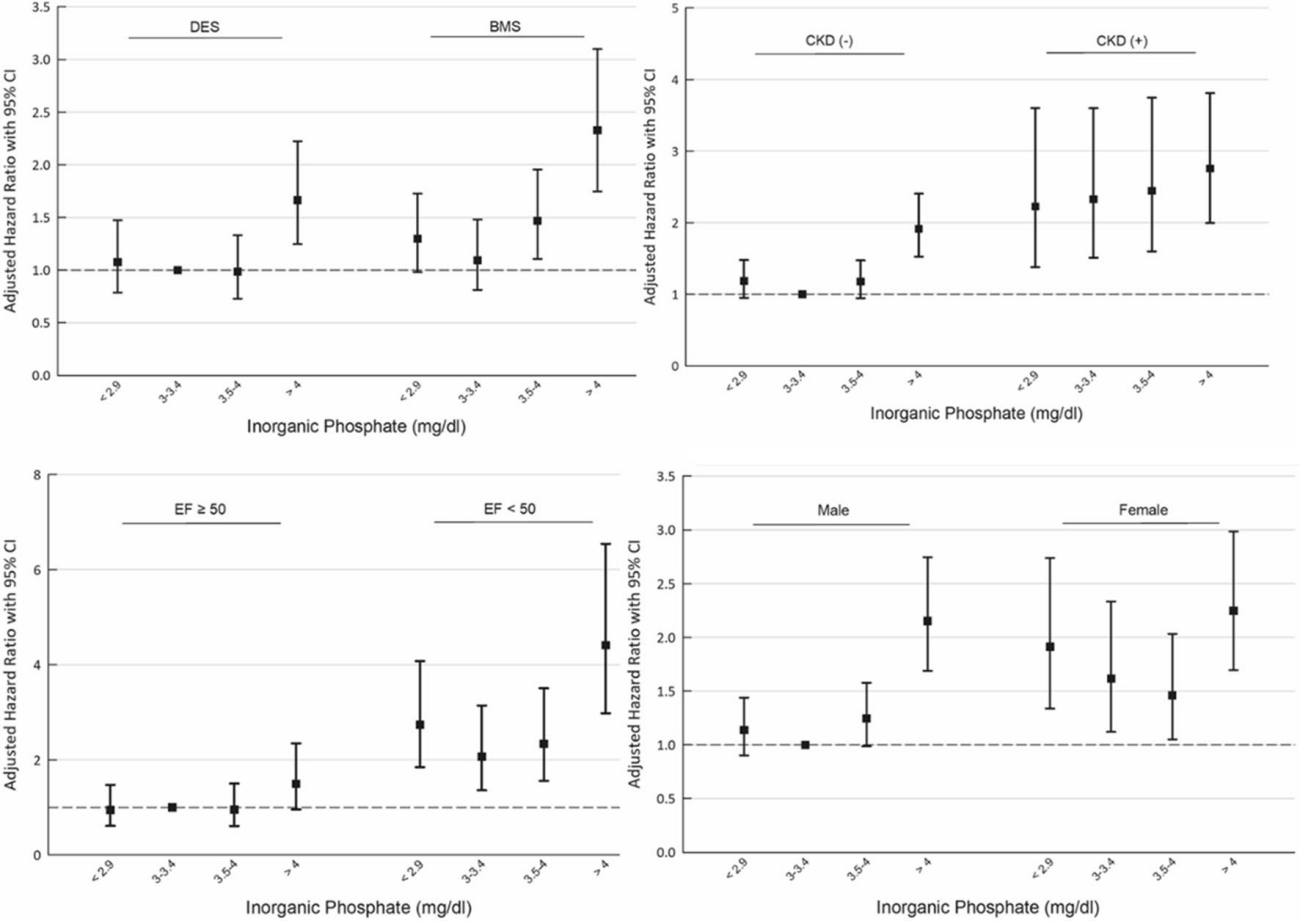
Prespecified subgroup analysis showing the relationship between serum phosphate quartile and the probability of the primary endpoint in different subgroups. The risk associated with phosphate level appeared more pronounced for male patients, patients received BMS, patients without CKD and patients with low EF. DES = drug eluted stents, BMS = bare metal stents, CKD = chronic kidney disease, EF = left ventricular ejection fraction.

Results of the prespecified subgroup analysis were demonstrated in Table 3, showing the risk associated with the phosphate level, which appeared different for several subgroups. A J-shaped association between poor outcome and phosphate level was more pronounced among the male gender, age > 65 years, using BMS, LVEF < 50%, eGFR < 60, LDL > 70 mg/dL, and the presence of MI at enrollment. (Fig. 3, supplement Fig. 2) The association between CV risk and phosphate level appeared to be linear for those with CKD.

**Table 3:**
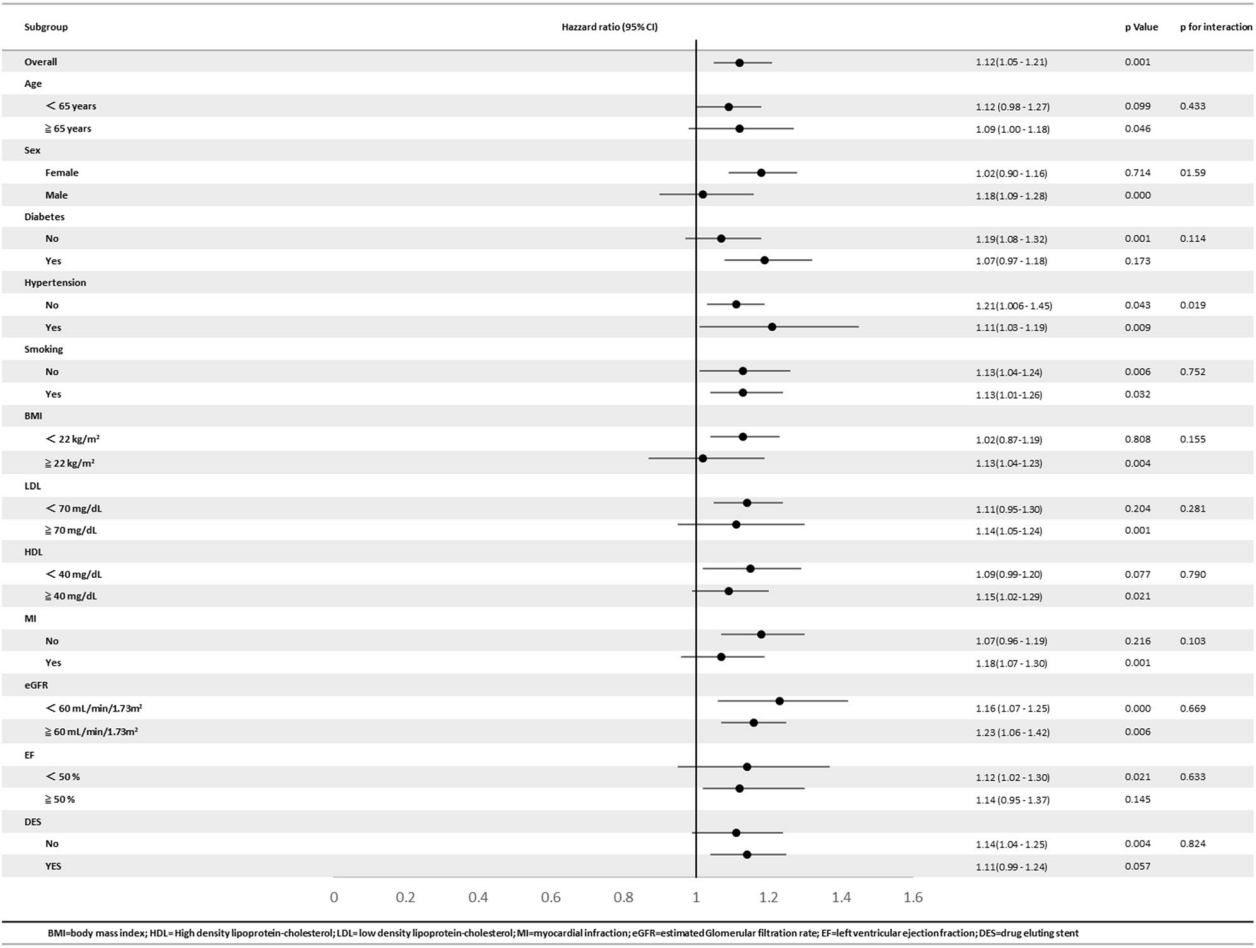
Subgroup analysis of phosphate and clinical outcome.

## Discussion

In this retrospective cohort study of 2894 Asian patients with CAD undergoing PCI, we demonstrated that (1) serum phosphate is an independent risk factor of adverse clinical outcomes after PCI; (2) the association between phosphate level and clinical outcomes is J-shaped, with the lowest future CV event risk observed for phosphate levels at approximately 3.0–3.4 mg/dL. Even adjusted for comorbidities and renal function, the serum phosphate level is independently associated with future risk of CV mortality, major CV events, and HF hospitalization. (3) Subgroup analyses further show the association between serum phosphate level and CV risk as more prominent in the male gender, age > 65 years old, using BMS, LVEF < 50%, eGFR < 60, LDL > 70 mg/dL with the presence of MI at enrollment; this indicates the importance of phosphate serum level in determining future CV outcomes in high-risk patients after a coronary PCI.

Phosphate homeostasis is essential for the maintenance of normal CV function. An abnormal phosphate balance and phosphate modulating hormones can damage the CV system. In cell and animal models, researchers found that phosphate plays a pivotal role in vascular calcification by inducing vascular smooth muscle cells (VSMCs) to undergo phenotypic changes in the osteochondrogenic phenotype^15,16^. Under the influence of phosphate, VSMCs lose the normal expression of calcification inhibitors and begin to express osteogenic markers^17^. A high phosphate level can also inhibit Vitamin D production. Without adequate vitamin D, the renin–angiotensin–aldosterone system activates in an unopposed manner, and results in dysregulated cardiomyocyte proliferation, inflammation, and atherosclerosis progression^18,19^. The association between phosphate exposure and calcification are both time- and dose-dependent, leading to diffuse medial calcification, the hallmark of CKD-related CV disease^16^. In addition to the cellular impact of phosphate to vessel walls, elevated serum phosphate is an independent risk factor for the development of CAD, progression of atherosclerotic plaques, MI, and CV death^2,4,5,20,21^. Phosphate dysregulation was observed to be associated with atrial fibrillation, left ventricular hypertrophy, heart failure, and stroke^22–25^. Moreover, many studies show the association between poor CV outcomes and phosphates, which are significant for patients without CKD, as the link extends beyond the commonly-recognized abnormal zone^4,21^. The Framingham offspring registry and the Cholesterol and Recurrent Events Study (CARE) registry show that for patients with normal renal function, phosphate levels > 3.5 mg/dL correlate with a higher risk of CV events^4,26^.

In the current study, subgroup analysis demonstrated the association of serum phosphate levels and future adverse outcomes in patients who underwent PCI; this showed that the J-shaped relationship was more significant in the male gender, age > 65 years, using BMS, LVEF < 50%, eGFR < 60, LDL > 70 mg/dL and the presence of MI at enrollment. Aronson et al. reported an independent association between serum phosphate, all-cause mortality, and heart failure in patients after acute myocardial infarction (AMI). The risk for mortality appears to increase with serum phosphate levels within the normal range and becomes even more prominent in the presence of CKD. Our current study expanded the association to all CAD patients, including those receiving elective PCI^27^. It also coincided with Kestenbaum’s observation that serum phosphate levels > 3.5 mg/dl were associated with a significantly increased risk of death^28^. Furthermore, our study reported a significant association between serum phosphate levels, higher risk of HF hospitalization, and total CV events (especially those using BMS, LVEF < 50%, eGFR < 60, LDL > 70 mg/dL and the presence of MI at enrollment); this suggests that higher phosphate carries a generally higher risk in high-risk patients. There is limited information explaining why a more prominent association between phosphate level and risk of total CV events was observed with BMSs than DESs. Although DES and BMS were reported to have no significant difference in the outcome of CV death or MI, DES use reduces the risk of revascularization and stent thrombosis^29^. Moreover, Sung et al. reviewed 966 patients receiving PCI in Taiwan and reported that DES was related to better outcomes (reducing MI and mortality) than BMS^30^. This may suggest that elevated serum phosphate adds more risk for vulnerable patients. A similar association between serum phosphate and future risk in the heart failure population was observed [42]. Our subgroup analysis provides insight about patients with high CV risk at baseline, particularly vulnerable to the additional risk from phosphate imbalance, and how intervention can reduce CV risk with DES use, while lipid lowering may mitigate these problems.

Our study has several limitations. First, this study was designed as a retrospective observation cohort: the timing and indication for phosphate level collection was not controlled. There is also potential for selection bias, as phosphate levels were probably only checked in patients with certain characteristics, such as renal function alteration or electrolyte imbalance. Second, we could only recruit patients receiving PCI, thus the association between phosphate and coronary artery bypass graft (CABG) was not explored. Third, we were unable to collect all information about medications and patient diets—which may have significantly affected phosphate levels. Forth, our study enrolled patients between 2006 to 2015, a time during which a large shift toward DES use in PCI have occurred in Taiwan. Thus, there may be some limitation in the generalizability of our results. However, in our subgroup analysis, DES use has no interaction with the prognostic value of serum phosphate. Fifth, we could only find 2984 patients who have a recorded phosphate level among the 8794 patient cohorts. In general, phosphate level is more frequently measured for patients who have higher risks. Thus, there may be some potential selection bias. Sixth, phosphate level can be influence by diet and medication (including diuretics) and may indicate co-existing factors such as dietary indiscretion or fluid overload. Thus, our results derived from pre-PCI phosphate level are best interpreted as correlation while causal association should be the subject of future studies.

In conclusion, our study showed that high serum phosphate levels are significantly associated with poor outcomes in CAD patients receiving PCI. The association between phosphate level and clinical outcome is J-shaped, with the lowest future CV risk observed for levels around 3.0–3.4 mg/dL after adjustment for comorbidities and renal function. The association between serum phosphate level and CV risk was more obvious in the male gender, age > 65 years, using BMS, LVEF < 50%, eGFR < 60, LDL > 70 mg/dL, and the presence of MI at enrollment, indicating the importance of serum level of phosphate in determining future CV outcomes in vulnerable patients after PCI.

## Supplementary Information

Supplementary Information.
