# Supplementary material for: Association between phosphate and long-term outcome in CAD patients underwent coronary intervention
Source: Sci Rep. 2021 Oct 11;11:20080. doi: 10.1038/s41598-021-99518-z (PMC8505547; doi:10.1038/s41598-021-99518-z)
Supplement: Supplementary file 1 — Supplementary Information. [file 41598_2021_99518_MOESM1_ESM.docx]

| Supplement Table 1  Factors associated with serum phosphate in multivariate linear regression | | | |
| --- | --- | --- | --- |
|  | Correlation Coefficient | 95% confidence interval | p value |
| Male Gender | -0.72 | -0.263~-0.66 | 0.01 |
| Age | -0.199 | -0.20~-0.13 | <0.001 |
| BMI | -0.003 | 0.889~-0.11 | 0.889 |
| Smoking (n, %) | -0.022 | -0.127~0.35 | 0.267 |
| Dyslipidemia (n, %) | 0.023 | -0.033~0.126 | 0.253 |
| Diabetes (n, %) | 0.024 | -0.035~0.147 | 0.231 |
| Hypertension (n, %) | 0.020 | -0.040~0.123 | 0.314 |
| Heart failure (n, %) | 0.031 | -0.19~0.176 | 0.113 |
| Stroke (n, %) | 0.043 | 0.021~0.294 | 0.024 |
| CKD (n, %) | 0.061 | 0.065~0.321 | 0.003 |
| ACS (n, %) | 0.024 | -0.030~0.127 | 0.223 |
| eGFR | -0.185 | -0.008~-0.005 | <0.001 |
| hemoglobin | -0.098 | -0.069~-0.024 | <0.001 |
| Uric acid | 0.098 | 0.029~0.068 | <0.001 |
| calcium | 0.114 | 0.109~0.220 | <0.001 |
| HDL | 0.018 | -0.002~0.005 | 0.353 |
| LDL | 0.031 | 0.000~0.002 | 0.116 |
| Beta blockers | -0.004 | -0.134~0.035 | 0.253 |
| ACEI/ARB | -0.025 | -0.134~0.035 | 0.253 |
| Statins | -0.023 | -0.132~0.041 | 0.298 |

BMI=body mass index, CKD=chronic kidney disease, ACS=acute coronary syndrome, HDL=high density lipoprotein cholesterol, LDL=low density lipoprotein cholesterol, ACEI/ARB= angiotensin converting enzyme inhibitor/ angiotensin receptor blockers

| Supplemental table 2  Improvement in discrimination performance and calibration for risk prediction of cardiovascular events in the multivariate-adjusted model after including phosphate level. | | | |
| --- | --- | --- | --- |
|  | C-index (95% CI) | NRI (95% CI) | IDI (95% CI) |
| Primary endpoint |  |  |  |
| Traditional risk factors | 0.61891 (0.5965-0.6413), p=reference | p= reference | p= reference |
| Traditional risk factors + phosphate | 0.6286 (0.6064-0.6509), p=0.0248 | 0.1825 (0.1011-0.2639), p<0.001 | 0.0042 (0.0017-0.0068), p=0.001 |
| MACE |  |  |  |
| Traditional risk factors | 0.58177 (0.5539-0.6096),  p= reference | p= reference | p= reference |
| Traditional risk factors + phosphate | 0.59524 (0.5672-0.6233), p=0.0757 | 0.1376 (0.0373-0.238), p=0.0075 | 0.0029 (0.0006-0.0052), p=0.0121 |

C-index=C-statistic index, CI=Confidence interval, IDI=integrated discrimination improvement, NRI= net reclassification improvement

| Supplemental table 3  Baseline clinical characteristics among selected and non-selected cohort. | | | |
| --- | --- | --- | --- |
|  | Selected  (n=2894) | Non-selected  (n=5810) | p value |
| Male Gender (n, %) | 2220 (74.4%) | 4923(84.73%) | <.0001 |
| Age(y/o) | 71.59±12.20 | 67.96±12.85 | <.0001 |
| BMI | 25.06±3.98 | 25.72±4.29 | <.0001 |
| Smoking (n, %) | 1149 (38.5%) | 1903(32.75%) | <.0001 |
| Dyslipidemia (n, %) | 696 (23.3%) | 619(10.65%) | 0.011 |
| Diabetes (n, %) | 1341 (44.9%) | 2107(36.27%) | <.0001 |
| Hypertension (n, %) | 2543 (85.2%) | 5113(88.00%) | 0.000 |
| Heart failure (n, %) | 593 (19.9%) | 565(9.72%) | <.0001 |
| Stroke (n, %) | 230 (7.7%) | 278(4.78%) | <.0001 |
| CKD (n, %) | 351 (11.8%) | 216(3.72%) | <.0001 |
| ACS (n, %) | 1318 (44.2%) | 1709(29.41%) | <.0001 |
| DES (n, %) | 1517 (50.8%) | 3625(62.39%) | <.0001 |
| Creatinine | 2.06±2.23 | 1.34±1.25 | <.0001 |
| eGFR | 53.20±29.34 | 66.60±24.29 | <.0001 |
| Hemoglobin | 12.23±2.12 | 13.15±1.84 | <.0001 |
| Uric acid | 6.60±2.08 | 6.41±1.75 | <.0001 |
| Calcium | 8.90±0.72 | 8.86±0.68 | 0.069 |
| HDL | 42.20±12.47 | 42.14±11.44 | 0.837 |
| LDL | 104.10±34.76 | 105.53±34.02 | 0.074 |
| Beta blockers | 1246 (41.8%) | 2646(45.54%) | 0.001 |
| Statins | 1386 (46.4%) | 3364(57.90%) | <.0001 |
| ACEI/ARB | 1378 (46.2%) | 2894(49.81%) | 0.001 |
| ACEI/ARB= angiotensin converting enzyme inhibitor/ angiotensin receptor blockers; DES=drug eluting stent; CKD=Chronic kidney disease, HDL= High density lipoprotein-cholesterol; LDL= low density lipoprotein-cholesterol | | | |

Supplement Figure 1.


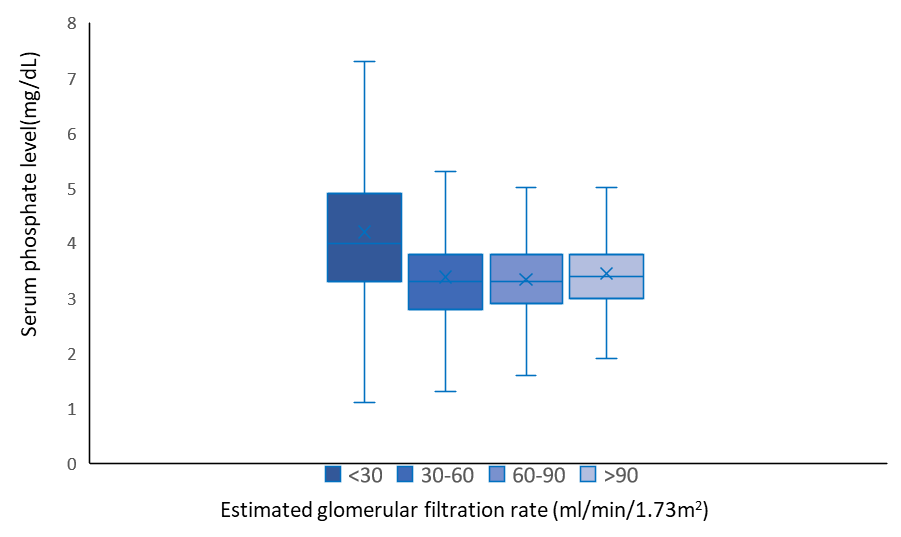


Supplement Figure 2.


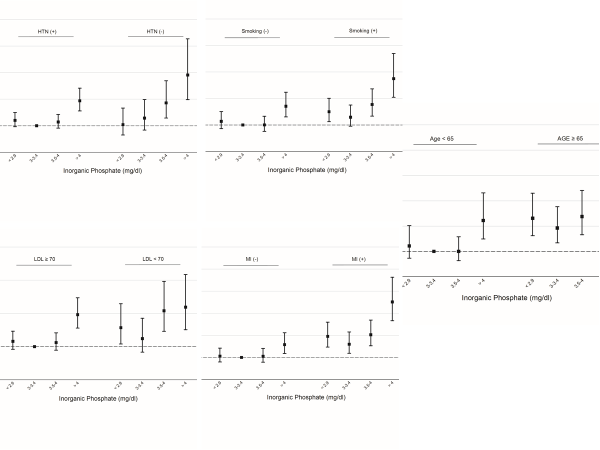


**Supplement Figure 1:** Relationship between serum phosphate and renal

function. Box–and–whisker plots of changes in serum phosphate

level according to the severity of renal dysfunction. The line within the

box denotes the median and the box spans the interquartile range

(25th to 75th percentiles). Whiskers extend from the 5th to 95^th^ percentiles.

**Supplement Figure 2:** Prespecified subgroup analysis showing the relationship between serum phosphate quartile and the probability of the primary endpoint in different subgroups. HTN=hypertension, LDL=low density lipoprotein cholesterol, MI=presented as acute coronary syndrome.

**Authors’ contribution:**

Tsung-Ying Tsai MD. ^1,2,3,6^, Pai-Feng Hsu MD.PhD. ^2.4.5.8^, Cheng-Hsueh Wu MD. ^4,7,8^, Ya-Ling Yang MD. ^7,8^,Su-Chan Chen MD. ^7,8^, Shao-Sung Huang MD. ^4,7,8^, Wan Leong Chan MD. ^4,5^, Shing-Jong Lin MD. ^4,5^, Jaw-Wen Chen MD. ^4,5^, Ju-Pin Pan MD. ^4,5,7^, Min-Ji Charng MD. ^4,5,7^, Ying-Hwa Chen MD. ^7,8^, Tao-Cheng Wu MD. ^7,8^, Tse-Min Lu MD.^7,8^, Po-Hsun Huang MD. ^7,8^, Hao-Min Cheng MD. ^4,7,8^, Chin-Chou Huang MD. ^4,7,8^, Shih-Hsien Sung MD.^7,8^, Yenn-Jiang Lin MD. ^5^, and Hsin-Bang Leu MD. ^1,2,4,5,7,8^

Concept/design^1^,

Data analysis/interpretation^2^,

Drafting article^3^,

revision of article^4^,

Approval of article^5^,

Statistics^6^,

Data collection^7^,

Other(logistic)^8^
